# Experimental Study on Mechanical Performance of Single-Side Bonded Carbon Fibre-Reinforced Plywood for Wood-Based Structures

**DOI:** 10.3390/ma18010207

**Published:** 2025-01-06

**Authors:** Krzysztof Szwajka, Joanna Zielińska-Szwajka, Tomasz Trzepieciński, Marek Szewczyk

**Affiliations:** 1 Department of Integrated Design and Tribology Systems, Faculty of Mechanics and Technology, Rzeszów University of Technology, ul. Kwiatkowskiego 4, 37-450 Stalowa Wola, Poland; m.szewczyk@prz.edu.pl; 2 Department of Component Manufacturing and Production Organization, Faculty of Mechanics and Technology, Rzeszów University of Technology, ul. Kwiatkowskiego 4, 37-450 Stalowa Wola, Poland; j.zielinska@prz.edu.pl; 3 Department of Manufacturing Processes and Production Engineering, Rzeszów University of Technology, al. Powstańców Warszawy 8, 35-959 Rzeszów, Poland; tomtrz@prz.edu.pl

**Keywords:** CFRP, modulus of elasticity, plywood, strain, wood-based materials

## Abstract

In addition to the traditional uses of plywood, such as furniture and construction, it is also widely used in areas that benefit from its special combination of strength and lightness, particularly as a construction material for the production of finishing elements of campervans and yachts. In light of the current need to reduce emissions of climate-damaging gases such as CO_2_, the use of lightweight construction materials is very important. In recent years, hybrid structures made of carbon fibre-reinforced plastics (CFRPs) and metals have attracted much attention in many industries. In contrast to hybrid metal/carbon fibre composites, research relating to laminates consisting of CFRPs and wood-based materials shows less interest. This article analyses the hybrid laminate resulting from bonding a CFRP panel to plywood in terms of strength and performance using a three-point bending test, a static tensile test and a dynamic analysis. Knowledge of the dynamic characteristics of carbon fibre-reinforced plywood allows for the adoption of such cutting parameters that will help prevent the occurrence of self-excited vibrations in the cutting process. Therefore, in this work, it was decided to determine the effect of using CFRP laminate on both the static and dynamic stiffness of the structure. Most studies in this field concern improving the strength of the structure without analysing the dynamic properties. This article proposes a simple and user-friendly methodology for determining the damping of a sandwich-type system. The results of strength tests were used to determine the modulus of elasticity, modulus of rupture, the position of the neutral axis and the frequency domain characteristics of the laminate obtained. The results show that the use of a CFRP-reinforced plywood panel not only improves the visual aspect but also improves the strength properties of such a hybrid material. In the case of a CFRP-reinforced plywood panel, the value of tensile stresses decreased by sixteen-fold (from 1.95 N/mm^2^ to 0.12 N/mm^2^), and the value of compressive stresses decreased by more than seven-fold (from 1.95 N/mm^2^ to 0.27 N/mm^2^) compared to unreinforced plywood. Based on the stress occurring at the tensile and compressive sides of the CFRP-reinforced plywood sample surface during a cantilever bending text, it was found that the value of modulus of rupture decreased by three-fold and the value of the modulus of elasticity decreased by more than five-fold compared to the unreinforced plywood sample. A dynamic analysis allowed us to determine that the frequency of natural vibrations of the CFRP-reinforced plywood panel increased by about 33% (from 30 Hz to 40 Hz) compared to the beam made only of plywood.

## 1. Introduction

The advantages of wood-based composites made from laminated wood, adhesives and other materials include better dimensional stability, more isotropic mechanical properties and higher durability compared to solid wood [1,2,3]. Wood-based materials exhibit more isotropic properties compared to solid wood. A plywood panel is a rigid structural material consisting of plywood veneers joined together so that the fibre orientation of the adjacent veneers is perpendicular. This change in fibre direction of the layers makes plywood stronger and causes it to exhibit more isotropic properties than solid wood. Wood-based composites are widely used in the construction, furniture, automotive and shipbuilding industries [1,4]. Wood-based materials are more homogeneous and isotropic compared to solid wood [5,6]. Wood-based composites are often used in structural applications as alternative materials to solid wood because they exhibit better dimensional stability and durability [2,5].

The most commonly used wood-based composites include sandwich materials, such as plywood or veneered wood, which are classified as layered laminates with better strength properties than natural raw materials [5,7,8]. In some applications, such as construction, wood-based materials are preferable to other engineering materials such as concrete, plastics or steel because it is a particularly important aspect to meet high-strength property requirements at a relatively low weight [6]. Plywood is a rigid structural material consisting of wood veneers glued together so that the veneer fibre orientation is perpendicular to the fibre orientation of the adjacent layer [1,3]. The properties of plywood plates depend on the type of wood used. In some cases, wood species (e.g., pine) are used for plywood production. However, they exhibit poor mechanical properties. Therefore, research is being conducted on how to improve these properties [9,10,11]. One option is to reinforce wood-based materials with different types of fibres with the main aim of improving their mechanical properties [12]. Wood-based materials with a hybrid structure have become necessary in engineering structures due to their better strength properties compared to basic materials [13,14]. Due to there being various interconnected materials with clearly different properties, it is necessary to analyse the resulting hybrid materials. Carbon fibre-reinforced polymer composites (CFRPs) are an interesting group of engineering materials [15,16]. Their main advantage, compared to conventional materials used in structural engineering, is a high specific strength (the ratio of tensile strength to specific gravity) [13,14,16]. Fibre-reinforced polymer materials have been widely regarded as good reinforcement materials in the repair and rehabilitation of cultural heritage buildings because of their good corrosion performance and high stiffness [17,18]. Most often, composite plates, rods or strips are used for repairing existing buildings or retrofitting recycled materials in historical buildings [19]. The use of CFRPs and glass fibre-reinforced plastics (GFRPs) for improving wooden columns provides the columns with increased compressive strength and improved ductility [20]. Many authors indicate a good retrofitting effect of using CFRP composites [21,22,23]. Due to CFRPs’ high specific strength, the use of CFRPs in vehicle construction has been steadily increasing in recent years, as exemplified by the increasingly widespread implementation of this material in many structural assemblies of motor vehicles and yachts.

The mechanical testing of polymer-reinforced wood-based materials ensure that these materials comply with performance requirements. The mechanical testing of composites includes static tests (in-plane shear, tensile, compression and three- and four-point bending tests) and dynamic (impact) tests. The advantage of the four-point bending test is that it provides a uniform bending moment between loading points [24]. The limitations of the three-point bending test are high shear stresses near supports and localised damage near the load point [25]. The last mentioned limitation is common to the four-point bending test. Tests should be adapted to the expected loads of the composites during operation [26]. The in-plane shear (IPS) test uses testing equipment designed for standard tensile testing. However, the IPS test is limited to in-plane shear properties. The impact test is used specifically to determine the impact strength and notch sensitivity of composites subjected to high-rate loading [27]. In fibre-reinforced polymers (FRPs) and hybrid wood-based composites, the impact properties depend on the properties of the fibres used for hybridisation, the interlaminar adhesion between the fibre and the matrix and the degree of densification of the wood-based materials [28,29]. In FRPs, the quality of the resin–fibre bond is examined based on interfacial and interlaminar shear strength [30]. A wide range of static tests adapted to the main types of loading of composite materials allows for the strength to be determined in conditions most similar to the operating conditions of the components [31]. Analytical analyses of composites operating under variable loads require determining the static mechanical parameters (i.e., modulus of elasticity). The strength properties of a CFRP composite depend primarily on the proportion of fibres in the composite, as well as on their weave [32]. The advantages of CFRP composites include high stiffness, vibration damping ability, high specific and fatigue strength, a low linear expansion coefficient and the ability to form complex shapes [33,34]. In addition, they are highly chemically resistant and do not absorb water [35]. Due to their characteristics, they are most often used for the construction of elements requiring high stiffness and resistance to harmful environmental effects [36]. Based on an analysis of the literature, it can be concluded that the main advantage of CFRP composites, in relation to conventionally used materials for construction, is greater mechanical strength at a significantly lower density [37]. With the reduction in economic and technological barriers related to this type of composite production, they are becoming an alternative to conventional construction materials [38]. The density of CFRP is, on average, 1.55 g/cm^3^ [39,40], while the density of plywood is 0.65 g/cm^3^ [2,41]. It can be seen that a CFRP panel is more than twice as dense as plywood. At the same time, the strength of CFRP panels is greater than strength of plywood. Therefore, it is beneficial to reinforce plywood as a base material with a small amount of CFRP. In recent years, various approaches have been employed to improve the properties of plywood and wood-based materials using CFRPs [14,15,16,42]. Bal et al. [43] investigated the mechanical properties of laminated veneer lumber reinforced with woven glass fibres. It was found that the reinforced samples showed a significant increase (213%) in the shear strength value compared to laminated veneer lumber. Auriga et al. [42] used carbon fibres as a reinforcement layer between plywood panels. The results of the tensile shear strength revealed that reinforcing plywood panels with carbon fibres increased both the modulus of elasticity and the modulus of rupture. Wang et al. [44] used CFRP and glass fibre-reinforced polymer to reinforce poplar laminated veneer lumber. They found that the use of reinforcement improved the modulus of elasticity and the modulus of rupture under vertical and horizontal loadings. Wei et al. [45] developed a theoretical model for the reinforcement of poplar laminated veneer lumber with CFRP. The modulus of elasticity was increased by 67% compared to unreinforced laminated panels. Omrani et al. [46] found that using kenaf fibres to reinforce plywood improved the modulus of elasticity of the resultant composites.

CFRP is gradually being used in the automotive, ship, railway and aviation industries due to its unique mechanical properties such as low density, high strength, high modulus of elasticity and a relatively simple design [47,48]. The ability to damp vibrations is one of the main parameters affecting the durability of the CFRP structure. Compared with traditional wood-based materials, CFRP has excellent vibration damping properties [49,50]. The requirements placed on current wood-based structural materials in modern engineering fields have led to the growth and development of the use of composite materials. The design flexibility, dimensional stability and other innumerable aspects that make composite materials superior have led to their wide application in aerospace, automotive and marine structures. An additional advantage is the formation of curvilinear structures, such as boat hulls, car structural modules, etc. Often, cutouts are made in such structures, which are often integral features of the laminated surfaces. The cutout reduces the mass of the laminated shell and changes the vibration characteristics. Moreover, the configuration of the structure plays a key role in controlling the stiffness. Although many researchers have conducted many studies on the strength of CFRP/wood joints, the published literature analysing the dynamic properties of such joints is very limited. No experimental dynamic analysis of such a material combination has been reported in the existing literature so far. Experimental analysis becomes extremely necessary to understand the dynamic behaviour in practical application. Therefore, an experimental modal analysis was conducted to obtain realistic dynamic characteristics of the sandwich joint. In this study, the effect of changing the neutral axis position during bending was also investigated. Knowing the exact location of the neutral axis allows for the precise determination of the strength parameters of the adhesives used in the joining process as well as the effect of internal defects on the strength of the obtained joint. The mechanical properties of wood elements can be significantly changed by the presence of natural defects (e.g., knots and cracks). Moreover, the shift in the neutral axis significantly affects the change in the values of the tensile and compressive stresses.

Each body or system of bodies (mechanism) has certain characteristic natural vibration frequencies, which depend on the shape of the body and the physical properties of the material. Free vibrations and natural vibrations of systems are extremely important in machine construction—obtaining knowledge of the natural vibration frequencies allows for the phenomenon of resonance to be avoided. Resonance in mechanical systems can be encountered, for example, when driving a car. The rattling of windows and covers and the vibrations and knocks coming from the chassis indicate that the natural vibration frequency of “rattling” and “knocking” components coincides with the current excitation frequency, i.e., with the engine vibration frequency for a given rotational speed. Loading structures or machines with excitations and with frequencies that coincide with the natural vibration frequency of the system are unacceptable. Applying a load with a vibration frequency equal to the resonance frequency of the machine or its elements will cause an uncontrolled increase in the amplitude of vibrations and will lead to the destruction of the structure. Therefore, in this article, research was undertaken in which a methodology for measuring and analysing the natural vibrations of structural elements made of plywood and CFRP-reinforced plywood panels was proposed. The previously mentioned construction materials were selected for analysis due to their most common use in the construction of yachts, sailing ships and campervans. This paper aims to conduct preliminary studies on the analysis of the mechanical properties of CFRP-reinforced plywood panels, which can be used to implement analytical or numerical models for design purposes. More specifically, in this study, static tension tests, three-point bending tests and dynamic analyses of CFRP-reinforced plywood hybrid plates are performed. The failure modes, stress–strain relationships, modulus of rupture and modulus of elasticity are analysed. Furthermore, the results obtained for CFRP-reinforced plywood laminates are compared with plywood as the reference material. Knowing the modal characteristics of construction materials allows for the vehicle construction process at the design stage to be carried out correctly.

## 2. Materials and Methods

### 2.1. Test Materials

In the tests, plywood panels (Biaform S.A., Białystok, Poland) produced from Scotch pine (*Pinus sylvestris* L.) veneers of 1.3 mm in thickness and 3 mm thick CFRP panel (DEXCRAFT, Warsaw, Poland) were used. The CFRP panel was glued on one side to the external surface of the plywood. For the three-point bending tests and static tensile test, samples with dimensions of 270 mm × 30 mm × 12 mm were used. For the dynamic tests, samples with dimensions of 500 mm × 30 mm × 12 mm were used. It should be emphasised here that both the transverse dimension of the sample and its length have an impact on the obtained characteristic of natural vibrations. It is only important to maintain identical dimensions of the tested element if the aim of the tests is to compare the dynamic properties of the structures. There are no strictly defined dimensions for the sample that should be used in similar tests.

The plywood panel used for glueing with the CFRP panel was 9 mm thick. For comparison purposes, a 12 mm thick plywood panel was used. In this way, the same thickness was maintained for the samples used for the subsequent comparative analysis. The properties of hybrid CFRP/plywood panels were compared with properties of reference plywood manufactured in accordance with the industrially available technology.

#### 2.1.1. Mechanical Properties of Plywood

**Figure A1:**
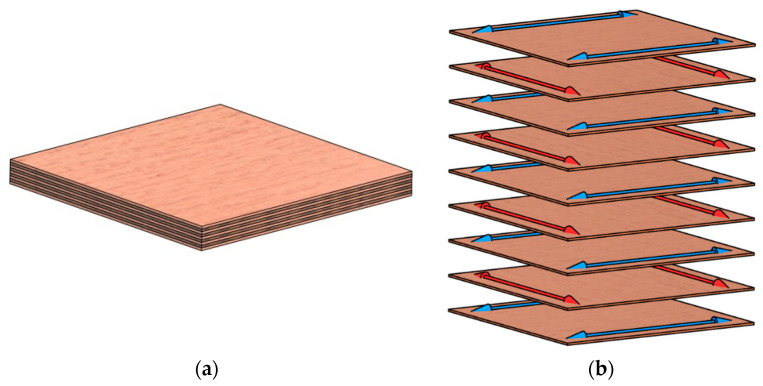
Plywood with 12 mm thickness: (**a**) panel and (**b**) arrangement of veneers.

Before the tests, the plywood samples (Biaform S.A., Białystok, Poland)were stored in the following conditions: a temperature of 22 ± 2 °C and 15 ± 2% humidity. Figure A1 shows a schematic representation of the alternating arrangement of veneers (0° and 90°) in the plywood used in the tests.

In the studies, it was assumed a priori that the additional material used to reinforce the plywood would be a CFRP panel glued at the top of the plywood. The CFRP plate with the same thickness is about 30–40% lighter than an element made of, for example, aluminium. If we add to this the practically zero thermal expansion of carbon fibres and the exceptionally attractive appearance of the material, it turns out that CFRP has many applications in industry, especially in final products to which this material gives a unique appearance. A panel made of 100% carbon fibres (so-called full-carbon fibre plate) is recommended, especially in construction applications where the high bending strength of the plate and its stiffness are beneficial. Examples of applications for such a panel include panels for building yachts, decorative interior elements, car panels and device casings. The top glossy layer of the panel is protected against UV. The other side of the CFRP panel is matte and rough. This feature was used in the fabrication of CFRP-reinforced plywood panels.

Fibre-reinforced polymers are used to strengthen wood-based composites, while the adhesive acts as a stress transferor [51]. In recent years, many types of fibres including carbon fibres, glass fibres, aramid fibres, basalt fibres and kevlar fibres were used as reinforcement materials in a polymer matrix [43,52]. CFRP laminates provide high strength, a high modulus of elasticity and exceptional fatigue resistance, and they are resistant to seawater and are thermally stable [53,54]. CFRP is a very lightweight material that is resistant to alkalis. Carbon fibre surfaces are non-reactive and non-polar [12]. The lower modulus of elasticity of glass fibres increases the strength of wood-based laminates to a greater extent [55]. The use of CFRP panels for the renovation of metal, concrete and wooden structures has become routine for many companies [56,57]. One of the greatest advantages of CFRP, apart from its favourable strength-to-weight ratio, is the decorative, modern appearance of its surface that is desired by customers.

At the stage of finishing a campervan, van, sailboat or yacht, where plywood is the basic finishing material, plywood is painted or laminated. In the configuration proposed in this article, there is no need to laminate or paint the top layer of CFRP/plywood panels later. The thickness of the proposed hybrid laminate is the same as that of typical plywood (12 mm) used as finishing material in transport vehicles. In the tests, single-sided reinforcement was used as this configuration is consistent with the objective of maintaining the aesthetics of the outer surface of the laminate for specific finishing applications while ensuring appropriate strength properties. A further increase in strength properties can be obtained by bonding CFRP panels on both sides of plywood. However, the increased cost of purchasing an additional CFRP board to reinforce plywood on both sides should be considered a limitation. Based on the strength tests carried out, it was found that single-sided bonding of CFRP laminate is the right compromise to achieve appropriate outer appearance, mechanical properties, weight of the panel and cost.

Before starting the main tests, measurements of selected mechanical properties of the materials used in the tests were performed. Modulus of rupture (MOR) was determined both parallelly and perpendicularly to the fibre direction of the outer veneer. Modulus of elasticity (MOE) was determined in directions parallel and perpendicular to the veneers. The experimental tests were carried out in accordance with the EN 310 standard [58]. Tests to determine the MOR and MOE were performed on a Zwick/Roell Z100 universal testing machine (Zwick/Roell, Ulm, Germany). Tests were repeated at least ten times, and average values of MOR and MOE were determined. The results of determining the mechanical properties of the plywood were statistically analysed using Statistica version 12 (StatSoft, Hamburg, Germany).

The static tensile test, carried out according to the EN 310 standard, is the basic and most frequently performed test to determine the strength of materials. The test consists of stretching a standardised sample until it breaks. The properties determined during the test include the ultimate tensile strength and modulus of elasticity.

Three-point bending test is one of the basic methods of determining the bending strength and modulus of elasticity. Three-point bending with a given speed of the punch movement is used, among other methods, to study the character of fracture and to determine the modulus of elasticity. According to the EN 310 standard, the loading speed should be selected so that the sample fails within 60 ± 30 s. The density of plywood panels was determined in accordance with EN 323.

#### 2.1.2. Mechanical Properties of CFRP

A CFRP laminate where the fibres in a plain-weave carbon fabric run at an angle of 0°/90° to each other was used. Thanks to the 1/1 weave, the fibres are perpendicular to each other. The CFRP panel (DEXCRAFT, Helenów, Poland)used in this study consisted of 50% carbon fibres and 50% epoxy resin. This increases the ratio of bending stiffness to compressive and tensile strengths. The precise fibre weaves allow for a very delicate fabric appearance, meaning that carbon products have an elegant design tailored to the needs of the interior design industry.

**Figure A2:**
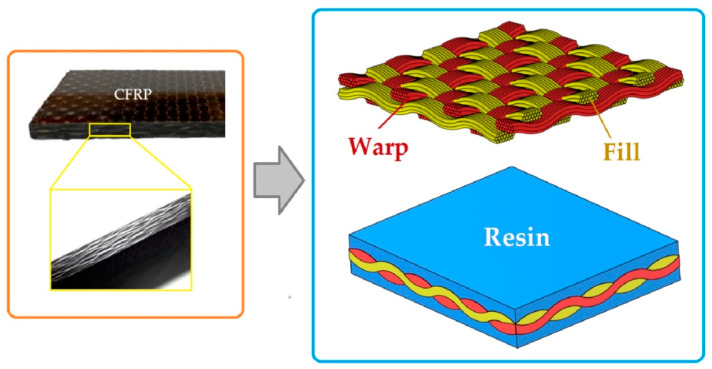
CFRP structure.

As shown in Figure A2, the specific weave structure, called the plain structure, contains interlacing longitudinal fibres (warp) and transverse fibres (filling) in an “over–under” arrangement, which results in a consistent and symmetrical cross pattern. The CFRP laminate used in this study was made of five layers, and the diameter of the carbon fibres was about 8 μm.

The CFRP panel is manufactured using prepregs, i.e., layers of carbon fibres that have already been saturated with epoxy resin at the stage of raw material production. The resin protects the fibres from damage and gives them the right viscosity, which facilitates the adhesion of the fabrics to the mould surface.

#### 2.1.3. Adhesive

**Figure A3:**
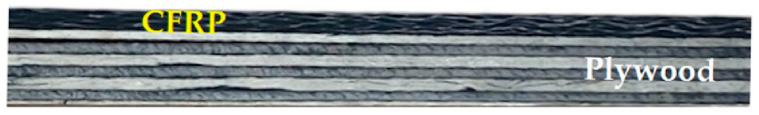
Cross section of CFRP-reinforced plywood (plywood was used as back material).

The plywood panel was glued to the CFRP panel using the ACRA LOCK SA 10-15 BLK methacrylic adhesive (Engineered Bonding Solutions, Titusville, FL, USA). According to the data sheet, this is an adhesive with increased flexibility recommended for joining composite panels in car trailers and campers. For laboratory purposes, the main selection criterion was the intended use of the adhesive and its efficiency. Economically, the prices of most similar adhesives are similar. The effectiveness of the reinforcement of the structural elements also depends on the correct execution of the glueing process. Before applying the adhesive, the surfaces of the veneer and the CFRP panel were sanded. Then, the CFRP panel and the plywood were properly cleaned using isopropyl alcohol (ELECTRO CHEM, Bydgoszcz, Poland). In the glueing process, in the first step, the adhesive layer was evenly applied to the plywood surface using an applicator (Engineered Bonding Solutions, Titusville, FL, USA) and then to the surface of the CFRP laminate. After applying the adhesive, the panels were pressed together to join them and to ensure precise adhesion of the glued surfaces. The joining parameters were as follows: temperature of 22 ± 1 °C, pressure of 0.35 N/mm^2^ and time of 6 h. The plywood samples were reinforced with a CFRP panel on one of its external surfaces. The Shore hardness measurement of the adhesive was performed according to the ASTM D2240 standard. This measurement was performed after the adhesive had completely cured. The curing time to full strength, as provided by the manufacturer, is 6 h. The hardness measurement was performed in the middle of the joint, between the plywood and the CFRP panel. Due to the fact that the adhesive is chemically cured, the place of hardness measurement (from the plywood or CFRP side) does not affect the changes in its value. Figure A3 shows a cross section of plywood reinforced with CRFP laminate that was prepared for testing.

### 2.2. Specimen Preparation

**Figure A4:**
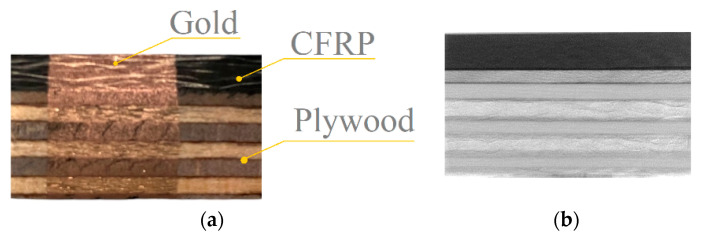
(**a**) Sample prepared for SEM analysis and (**b**) X-ray micrograph of CFRP-reinforced plywood laminate.

The microstructure and the chemical composition of the CFRP-reinforced plywood panels was analysed using a TESCAN^®^ scanning electron microscope (TESCAN, MIRA3, Brno, Czech Republic). In addition, the sample of the panel was X-rayed and photographed using a General Electric phoenix v|tome|xm X-ray tomograph (TESCAN, MIRA3, Brno, Czech Republic). Before starting the analysis using a scanning microscope, it was necessary to properly prepare the samples. Due to the fact that plywood belongs to the category of dielectrics, it was necessary to sputter a thin layer of electrically conductive material (gold) (PIK INSTRUMENTS sp. z o.o., Piaseczno, Poland) on its surface (Figure A4). The methodology of the sputtering process is described in detail in [2].

### 2.3. Measurement Campaign

As part of the tests, in addition to the three-point bending test, the deformations occurring in the bent sample were analysed. In addition, the dynamic characteristics of the obtained CFRP-reinforced plywood panel were determined.

#### 2.3.1. Three-Point Bending Test

**Figure A5:**
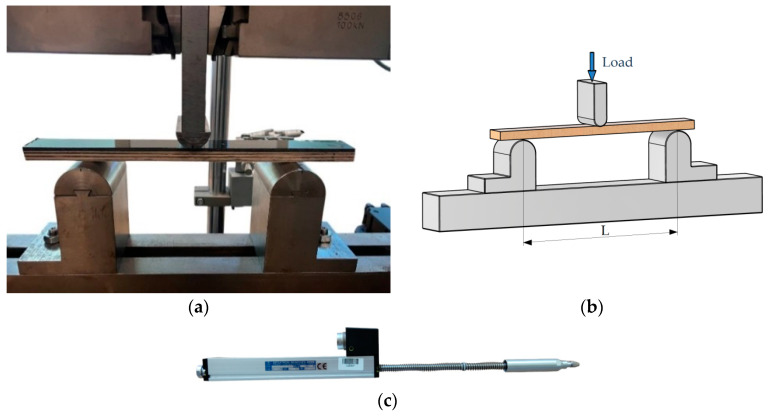
Three-point bending test: (**a**) measuring stand, (**b**) measurement scheme (L = 240 mm) and (**c**) Megatron SPR18-100 sensor for measuring linear displacements.

In the three-point bending test (Figure A5), conducted according to the EN 310 standard, a cuboid-shaped specimen is placed on two supports and subjected to continuous deformation by means of a centrally placed bending punch using a Zwick/Roell Z100 testing machine (Zwick/Roell, Ulm, Germany). During the deformation process, the force exerted by the bending punch and the deflection of the specimen under the bending punch are registered.

Plywood and a CFRP/plywood panel were used for the three-point bending tests on a universal mechanical testing machine according to the EN 310 standard. Each sample was tested in pure bending conditions with a span of L = 240 mm and a punch speed of 0.3 mm/min. The values of MOE and MOR were determined. The deflection at mid-span (Δ) was measured using a Megatron SPR18-100 linear displacement sensor (MEGATRON Elektronik GmbH & Co. KG., Munich, Germany). The tests were carried out according to the EN 310 standard.

**Figure 1:**
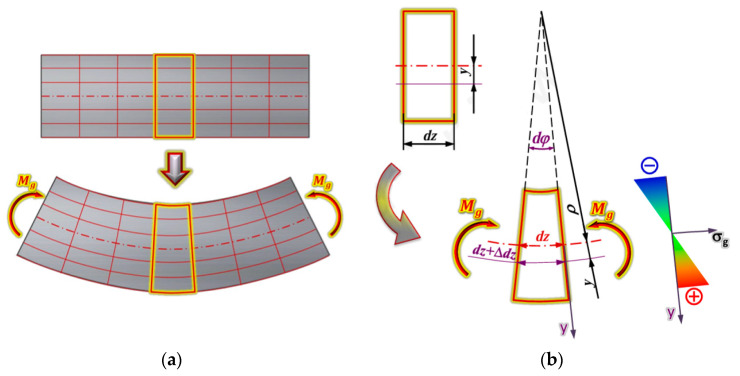
Deformations of a bent beam: (**a**) deformations in the longitudinal direction and (**b**) deformations on the beam’s cross section.

Figure 1 shows a sample of a rectangular cross section with a grid of lines parallel to the beam’s plane of symmetry and perpendicular to that plane.

Yellow indicates the mesh elements located in one plane of the cross section of the sample in the undeformed state (Figure 1a). After the beam in the symmetry plane “yz” is deformed by the action of the moment, M_g_, the planes of the marked cross section will tilt towards each other at an angle. The upper and lower fibres will shorten and lengthen, respectively. The length of the fibres located in the neutral plane will not change. Under the influence of the moment, M_g_, the sample will expand in the upper part, and the initial rectangular cross section of the sample will take a trapezoid shape (Figure 1b). If we denote the radius of curvature of the neutral layer by ρ, then the length of the neutral layer fibre section can be expressed by the following formula:

(1) dz=dφ·ρ

The fibres located at a distance, y, from the neutral layer had a length equal to dφ·ρ before deformation, whereas after deformation,

(2) $$dz+∆dz=\left(\rho +y\right)d\varphi$$

The relative elongation of the fibre is therefore

(3) $$\varepsilon =\frac{\left(dz+∆z\right)-dz}{dz}=\frac{\left(\rho +y\right)d\varphi -\rho \cdot d\varphi }{\rho \cdot d\varphi }$$

After simplification, we obtain

(4) $$\varepsilon =\frac{y}{\rho }$$

**Figure 2:**
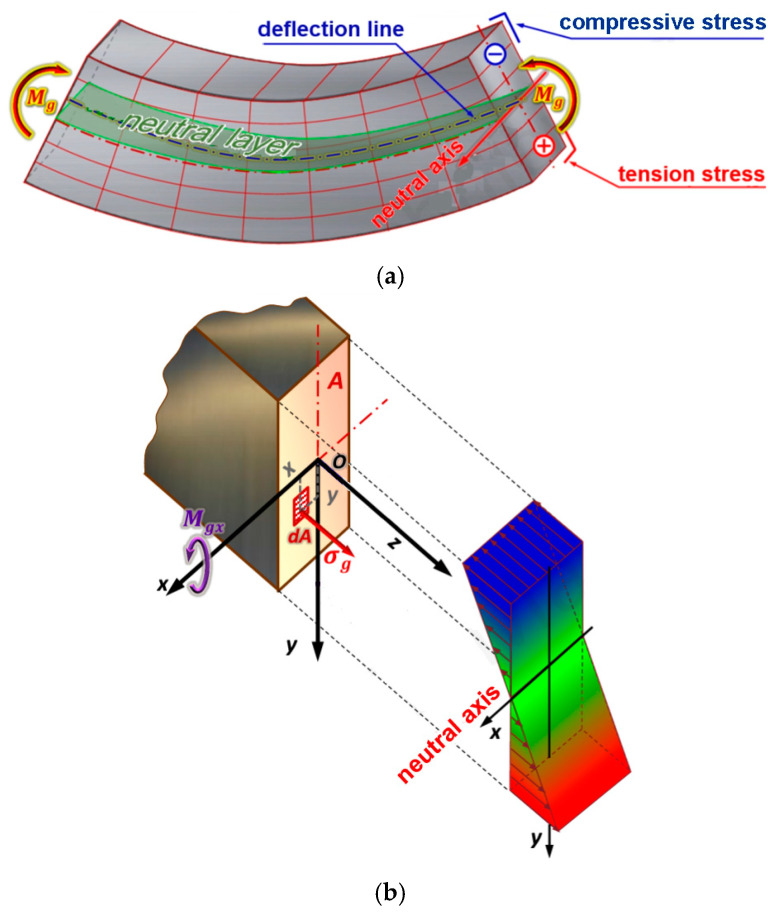
(**a**) A diagram of the stress state and (**b**) stress distribution in the beam.

For a given cross section, the radius of curvature ρ of the bent beam is constant (ρ = const); therefore, the above formula shows that the stresses at individual points of the cross section of the bent beam change proportionally to the distance of these points from the neutral layer, as shown in Figure 2b.

According to Hooke’s law,

(5) $$\sigma_{g}=E\cdot \varepsilon \to \sigma_{g}=\frac{E}{\rho }\cdot y$$

The moment of the elementary force in relation to the neutral axis (Figure 2b) is $\sigma_{g}\cdot y\cdot dA$, and the sum of these moments integrated over the entire area (A) of the cross section of the beam must balance the moment (M_gx_) applied to the beam, so the second equilibrium condition of the beam under consideration (Figure 2) takes the following form:

(6) $$M_{gx}=\int_{A}^{u}\sigma_{g}\cdot y\cdot dA=\frac{E}{\rho }\cdot \int_{A}^{u}y^{2}\cdot dA$$

The obtained integral is the moment of inertia of the cross section of the beam l_x_:

(7) $$M_{gx}=M_{g}=\frac{E}{\rho }\cdot I_{x}$$

Therefore, Equation (7) can be written in the following form:

(8) $$\frac{1}{\rho }=\frac{M_{g}}{E\cdot I_{x}}$$

and after substituting Equation (5),

(9) $$\sigma_{g}=\frac{E}{\rho }\cdot y\to \frac{1}{\rho }=\frac{\sigma_{g}}{E\cdot y}$$

(10) $$\frac{\sigma_{g}}{E\cdot y}=\frac{M_{g}}{E\cdot I_{x}}\to \sigma_{g}=\frac{M_{g}}{I_{x}}\cdot y$$

#### 2.3.2. Cantilever Bending Test

**Figure A6:**
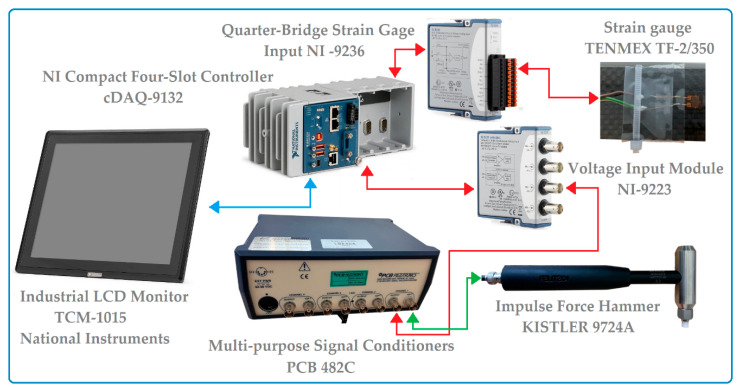
Measuring track for analysis of deformations and dynamic characteristics.

In the tests, in addition to the three-point bending test, cantilever bending tests were carried out assuming that a cantilever beam was fixed on one side. The other end of the beam was free. Such a test was conducted with the aim of obtaining a more convenient measurement of sample deformations ε and, above all, the possibility of conducting a dynamic analysis of the sample. During the bending test, deformation signals ε were recorded in the direction perpendicular to the plane of the CFRP-reinforced plywood panel. A schematic diagram of the measurement track configuration and the measurement data archiving system from the dynamic analyses of the beam is shown in Figure A6.

**Figure A7:**
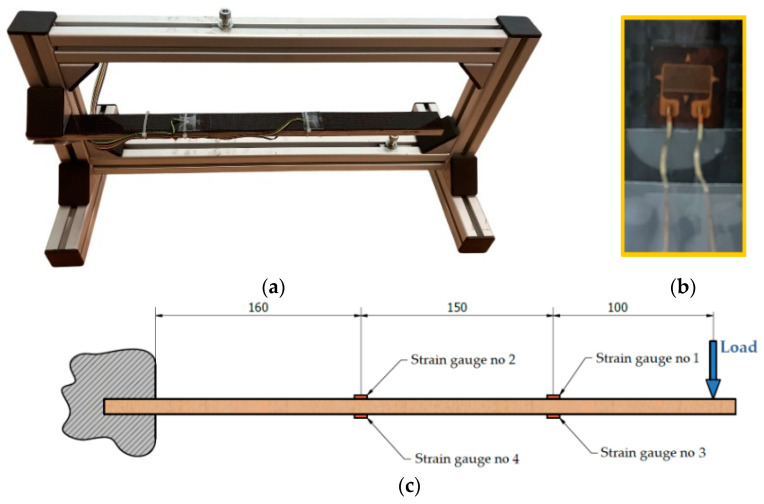
Measurement of cantilever beam deformations: (**a**) measuring stand, (**b**) strain gauge and (**c**) measurement scheme.

The deformation values of the samples during cantilever bending were measured using TENMEX T-2/350 strain gauges (TENMEX, Łódź, Poland), which were glued to the beam according to the configuration shown in Figure A7. According to TENMEX recommendations, the strain gauges were glued to the CFRP/plywood surface using a room-temperature curing polyester glue. After thoroughly cleaning the surface, the typical procedure for glueing a strain gauge was used. The strain gauges were connected to the circuit to form a quarter Wheatstone bridge circuit.

During the tests, deformation measurements were taken for both the sample obtained from the combination of plywood with a CFRP panel and the plywood only. The force loading of the beam shown in Figure A7 was 30 N. The strain gauges were glued to both sides of the bending beam. The signal recording system used the NI Compact Four-Slot cDAQ-9132 Controller (National Instruments, Austin, TX, USA). The signals from the strain gauges were registered via the quarter-bridge strain gauge NI-9236 (National Instruments, Austin, TX, USA).

**Figure A8:**
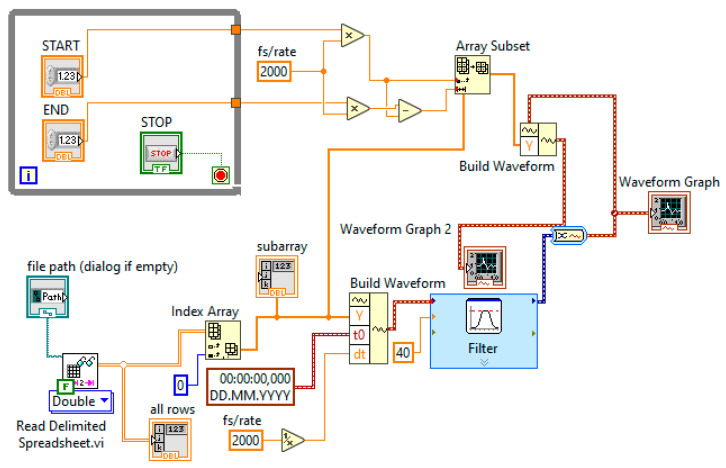
A block diagram of application for the determination of the natural frequency of sample vibrations in LabVIEW 2022.

Important information regarding the dynamic properties is the frequency characteristics of the analysed beam, that is, the beam’s response to the excitation F(t). In order to excite beam displacement, a KISTLER 9724A modal hammer (KISTLER, Winterthur, Switzerland) was used. The response of the system to excitation was determined based on the deformations of the tensometric system. For this purpose, an application was developed in the LabVIEW 2022 environment that allows for the determination of the natural frequency of sample vibrations based on the recorded deformation signals. The diagram of the developed application is shown in Figure A8.

#### 2.3.3. System Dynamics

An important stage in the dynamic analysis of a laminate is determining its frequency response. The Dirac impulse (called the Dirac delta function) was used to determine the impulse response of the system.

A dynamic analysis of a CFRP-reinforced plywood sample and an unreinforced one was performed. For this purpose, the system was excited with excitation in the form of a Dirac impulse using a modal hammer. Knowing the excitation force signal and the system response in the form of deformation, a modal analysis was performed to determine the natural frequency of the sample (Figure A7a). The methodology for determining the frequency response is described in detail in [59].

**Figure 3:**
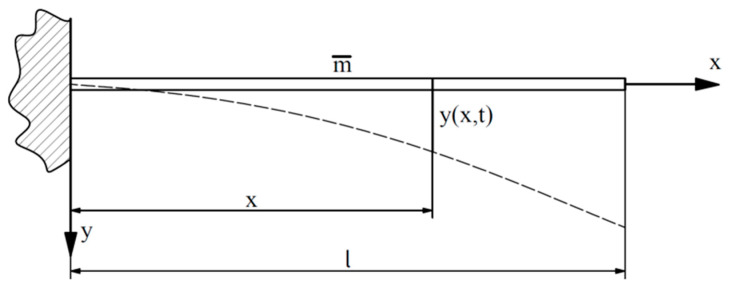
A cantilever beam with the mass, m, distributed evenly along the entire span, l ($\bar{m}=m/l)$.

During the vibration movement of the sample shown in Figure A7 and Figure 3, the displacements, y, of its arbitrary point are the functions of two independent variables, the abscissa, x, and time, t:

(11) y=f(x,t)

**Figure A9:**
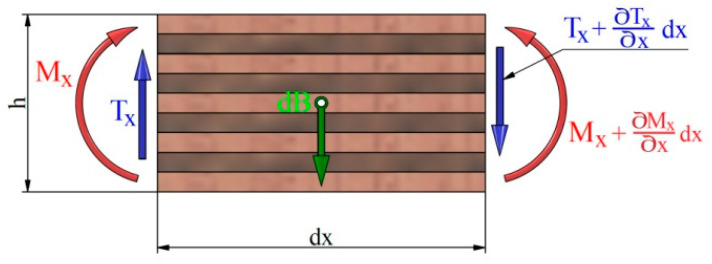
Equivalent loads acting on the considered element of infinitesimal length, dx.

Since the displacement, y, is defined in relation to the position of static equilibrium, when considering the forces acting on the beam, its deadweight is ignored, and only the forces of inertia and elasticity are taken into account. Considering small vibrations, i.e., those that can be described by linear differential equations, in the main bending plane, the displacements are perpendicular to the longitudinal axis, x, and the dimensions of the beam’s cross section are small compared to its length (l/h > 10). During the vibration motion, each beam element is subjected to the action of inertia forces caused by changes in the velocity of the motion (acceleration). The equivalent loads acting on the considered element of infinitesimal length, dx, are shown in Figure A9.

In addition, the elementary fragment of the beam is subjected to an inertia force equal to

(12) $$dB=-\bar{m}\frac{\partial^{2}y}{\partial t^{2}}dx$$

where $\bar{m}=m/l$.

The introduction of partial derivatives is forced by the dependence of the displacement in the y direction on two variables (Equation (12)). After determining the inertia force according to d’Alembert’s principle, the equilibrium conditions can be derived. Therefore, the condition for the sum of the projections of forces on the vertical axis is as follows:

(13) $$\frac{\partial T_{x}}{\partial x}dx+dB=0$$

After substituting the dB values in Equation (13) and dividing by dx, we obtain

(14) $$\frac{\partial T_{x}}{\partial x}-\bar{m}\frac{\partial^{2}y}{\partial t^{2}}=0$$

Then, the relationship between transverse force and bending moment is used:

(15) $$\frac{\partial M_{x}}{\partial x}=T_{x}$$

The differential equation of the axis of a bent beam is as follows:

(16) $$\frac{\partial^{2}y}{\partial x^{2}}=-\frac{M_{x}}{EJ}$$

After introducing the above-mentioned relations into Equation (13) and arranging them, we obtain a fourth-order partial differential linear equation:

(17) $$\frac{\partial^{2}y}{\partial t^{2}}+\frac{EJ}{\bar{m}}\frac{\partial^{4}y}{\partial x^{4}}=0$$

This is the equation of free transverse vibrations of a beam with a uniformly distributed mass, neglecting the influence of transverse forces and inertial forces in rotational motion, which would give the moment of inertia relative to the z axis, perpendicular to the x and y axes of the beam under consideration. This limited our analysis to searching only for such solutions of Equation (17) that define the so-called “standing wave” [60]. In such a case, the particular solution of the described equation can be presented as a function of separated variables, i.e., in the form of a product of two functions (Fourier method). The first function depends only on the abscissa, x, and the second only on time, t:

(18) $$y=X\left(x\right)T(t)$$

where the function X(x) determines the type of natural vibrations and is called the natural function of the vibration form of a given system, while T(t) is a function of time. It should not be confused with the transverse force T_x_. The partial derivatives of Equation (17) will be written in the following form:

(19) $$\frac{\partial^{2}y}{\partial t^{2}}=X(x)\frac{d^{2}T(t)}{dt^{2}}$$

(20) $$\frac{\partial^{4}y}{\partial x^{4}}=T(t)\frac{d^{4}X(x)}{dx^{4}}$$

Expressing the partial derivatives of function y in terms of ordinary derivatives and substituting Equations (19) and (20) into Equation (17) results in the following:

(21) $$X\left(x\right)\frac{d^{2}T\left(t\right)}{dt^{2}}+\frac{EJ}{\bar{m}}T(t)\frac{d^{4}X(x)}{\partial x^{4}}$$

hence,

(22) $$\frac{EJ}{\bar{m}}\frac{\frac{d^{4}X(x)}{\partial x^{4}}}{X(x)}=-\frac{\frac{d^{2}T\left(t\right)}{dt^{2}}}{T(t)}$$

From the presented relation, it follows that the left side depends only on the abscissa, x, and the right side only on the time, t. We can therefore conclude that both the left and the right sides of Equation (22) do not depend on the variables x and t, that is, each of them is equal to a constant number, which we will denote as ω_2_. This conclusion also follows from the fact that Equation (22) holds at every point of the beam and at every moment of time. In this way, instead of one differential equation with partial derivatives of the function y(x, t), we obtained two independent, ordinary homogeneous differential equations:

(23) $$\frac{d^{2}T\left(t\right)}{dt^{2}}+\omega^{2}T\left(t\right)=0$$

(24) $$\frac{d^{2}X\left(x\right)}{dt^{2}}-\omega^{2}\frac{\bar{m}}{EJ}X\left(x\right)=0$$

These equations have two solutions.

The first solution is

(25) $$T(t)=A\sin(\omega t+\lambda_{0})$$

which proves that the considered motion is an oscillatory motion with frequency ω.

The second solution is

(26) $$X\left(x\right)=C_{1}sin\alpha x+C_{2}cos\alpha x+C_{3}sinh\alpha x\;C_{4}cosh\alpha x$$

which describes the form of oscillations.

In Equation (26), the following notation was introduced:

(27) $$\alpha =\sqrt[{4}]{\frac{\bar{m}\omega^{2}}{EJ}}$$

The constants C_1_, C_2_, C_3_, and C_4_ were determined, among others, on the basis of the boundary conditions, i.e., depending on the method of beam attachment.

At the support (x = 0), both beam deflection, y_x_, and the angle of rotation, ψ_x_, are equal to zero, while for the free beam end (x = l), the bending moment, M_x_, and the transverse force, T_x_, are equal to zero:

(28) $$x=0{,y}_{x}=X\left(x\right)=0,\psi_{x}=\frac{dX(x)}{dx}=0$$

(29) $$x=l{,M}_{x}=-EJ\frac{d^{2}X(x)}{dx^{2}}=0,T_{x}=-EJ\frac{{dX}^{3}(x)}{dx^{3}}=0$$

After substituting Equation (26) into Equations (28) and (29), the relationship between the constants is obtained:

(30) $$C_{2}+C_{4}=0,C_{1}+C_{3}=0$$

Substituting Equation (30) into the solution of Equation (26) will give two homogeneous equations for the third and fourth conditions (Equations (28) and (29)). In turn, equating the determinant of these equations to zero, as a necessary and sufficient condition for the existence of a solution, will lead to the following relationship:

(31) cos⁡αl·cosh⁡αl=−1

We can find the roots of this equation, for example, graphically, by plotting the functions cos⁡αl and $\frac{1}{cosh\alpha l}=\frac{2}{e^{\alpha l}+e^{-\alpha l}}$. The intersection points of these graphs will give the following values of α:

(32) $$\alpha_{1}=\frac{1.875}{l},\alpha_{2}=\frac{4.694}{l},\alpha_{2}=\frac{7.855}{l},etc.$$

The relationship between the natural frequency and the value of α is defined by Equation (27). The calculated vibration frequencies of the beam, counting from the least basic one, are as follows:

(33) $$\omega_{1}=\frac{3.516}{l^{2}}\sqrt[{u}]{\frac{EJl}{m}},\omega_{2}=\frac{22.034}{l^{2}}\sqrt[{u}]{\frac{EJl}{m},}\omega_{3}=\frac{61.701}{l^{2}}\sqrt[{u}]{\frac{EJl}{m}},etc.$$

## 3. Results and Discussion

### 3.1. EDS Analysis of Carbon Fibre-Reinforced Plywood

**Figure 4:**
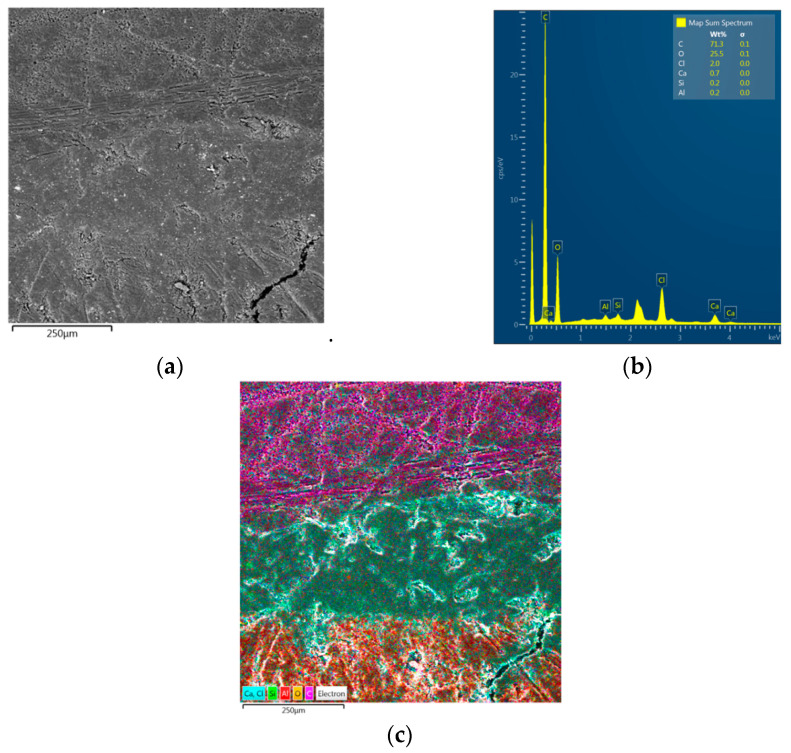
(**a**) SEM micrograph of interface between CFRP laminate, glue and plywood; (**b**) EDS spectrum and (**c**) EDS layered images for area shown in (**a**).

**Figure 5:**
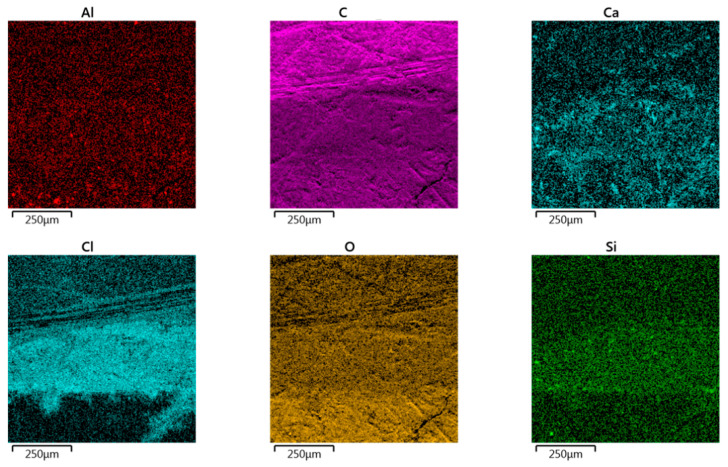
EDS elemental mapping in the area shown in Figure 1a.

Figure 4b presents the results of the chemical composition of the sample shown in Figure A4. An interface between the CFRP laminate, adhesive and plywood (from top to bottom in Figure 4c) was considered. As can be seen (Figure 4c), there is a varied distribution of elements in the analysed area. As can be predicted, when analysing the area of the CFRP panel, the largest share of carbon occurred here. This is understandable due to the structure of the CFRP panel. Moving downwards, we can distinguish the glue layer, for which a significant composition of chlorine and silicon is noted. This is the result of the participation of these elements in the chemical composition of the adhesive. In the last layer of the analysed area, the dominant elements are carbon and oxygen. This is obviously due to the fact that plywood is an organic material. Figure 5 shows the distribution of dominant elements in the analysed area (Figure 4a).

### 3.2. Mechanical and Physical Properties of Test Materials

Table 1 summarises the selected mechanical and physical properties of the plywood panel and CFRP panel used in the tests.

**Figure A10:**
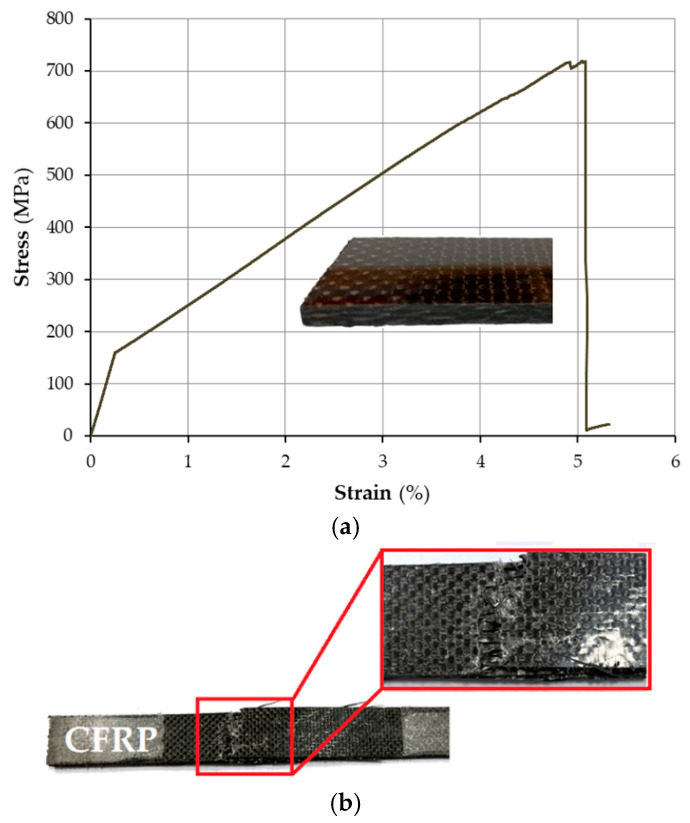
(**a**) Stress–strain tensile curve for CFRP panel and (**b**) sample after destruction.

**Figure A11:**
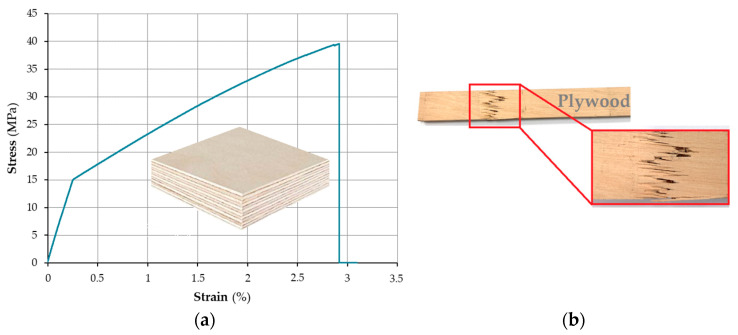
(**a**) Stress–strain tensile curve for 12 mm thick plywood and (**b**) sample after destruction.

Figure A10 presents an example of a stress–strain curve obtained in the static tensile tests of the CFRP panel. Figure A11 presents one of many stress–strain graphs obtained in the static tensile test.

When comparing the results of the studies (Table 1), it can be seen that the materials used in the studies are characterised by significantly diverse mechanical properties. The mechanical properties of the materials are of fundamental importance for understanding the behaviour of components in engineering structures. These properties play a key role in the design of many structures.

### 3.3. Three-Point Bending Test

**Figure A12:**
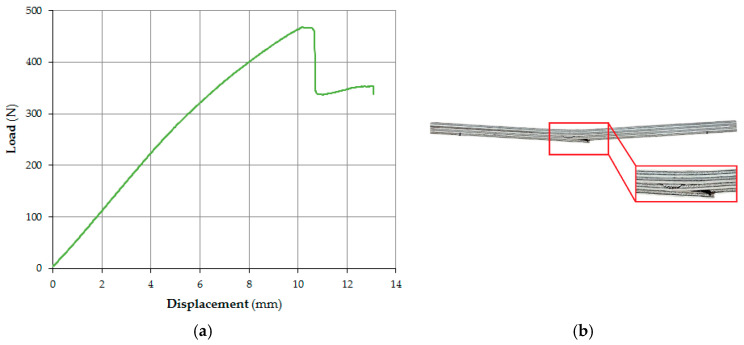
(**a**) Load–displacement curve (three-point bending test) for 12 mm thick plywood and (**b**) sample after failure.

From the obtained Equation (11), we can see that in a sample, the stresses in the cross section are directly proportional to the distance, y, of a given point of the cross section from the neutral layer, as shown in Figure A12. According to Equation (10), in a bent beam, the greatest stresses occur in the fibres located farthest from the neutral axis. In the case when the neutral axis is also the axis of symmetry of the sample, the greatest stresses occur in the lower fibres (tensioned) and in the upper fibres (compressed).

**Figure A13:**
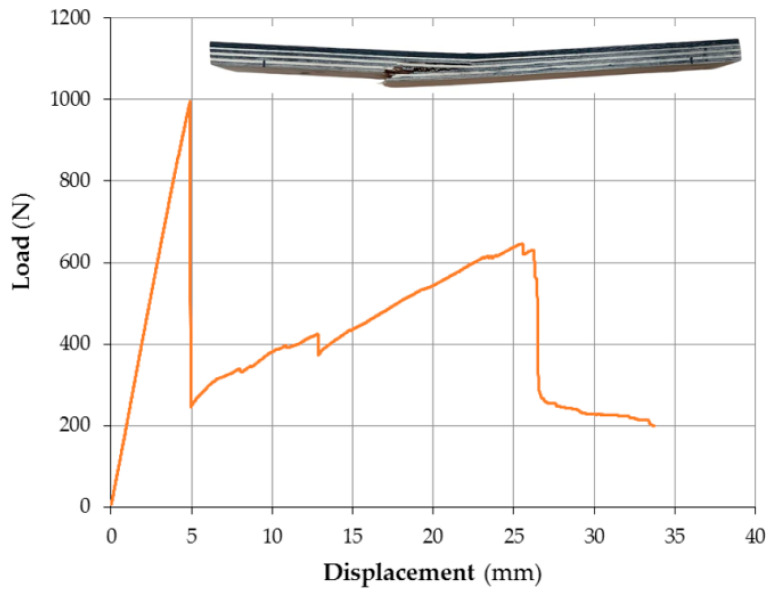
Three-point bending test results of CFRP-reinforced plywood sample.

All samples used in the three-point bending test failed in the areas furthest from the neutral axis. In the case of samples not reinforced with the CFRP panel, the failure was violent (Figure A12) and manifested as a sudden crack. In the case of the CFRP-reinforced samples, the failure process was relatively slow with an observed crack propagation (Figure A13). During the bending tests, no failure of the adhesive joint between the CFRP panel and plywood was observed.

Selected properties of the adhesive are listed in Table 2.

**Figure 6:**
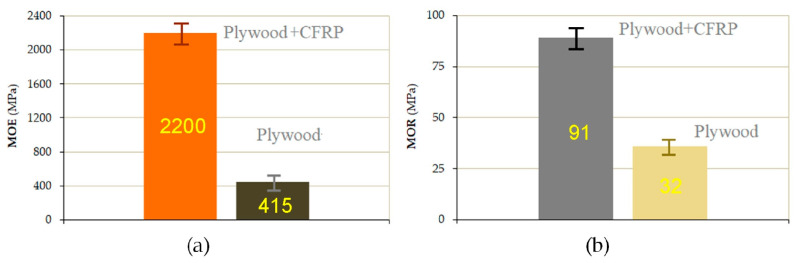
(**a**) MOE and (**b**) MOR values obtained in three-point bending test.

The failures of the CFRP-reinforced plywood samples in the bending test can be classified as plastic fracture damage. After reaching the maximum force, the force value decreased rapidly due to damage to the plywood layers and the decrease in the overall stiffness of the sample. At a later stage, the force value started to increase again due to the increasing resistance of the CFRP layer against deformation. Macroscopic failure (crack) in the sample occurred at a displacement of the punch of about 25 mm. Compared to the CFRP panel, which is subjected to compression in the first stage of the bending process, the tensile layer (plywood) becomes the weakest area of the entire sample, and brittle fracture damage was observed in this region. The loading force of the plywood sample did not decrease significantly, indicating that the fracture of the sample mainly manifested as the sudden brittle fracture of the plywood layers from the tensile side. The use of a CFRP panel to reinforce the compression zone of the samples effectively reduced the deformation of the outer layers of plywood subjected to tensile stress. Based on the analysis of the deformation of the plywood in the mid-span of the samples, it was found that deformation (and therefore the tensile stress) was reduced in the lower layers of the CFRP-reinforced plywood samples compared to the unreinforced plywood. In the CFRP-reinforced plywood samples, the compressive and tensile stresses in the plywood were effectively reduced. In the static three-point bending test of the CFRP/plywood hybrid samples, there was an obvious shift in the neutral axis toward the CFRP panel. Significant increases in the MOR and MOE values (Figure 6) were observed for the CFRP-reinforced samples. The use of CFRP panel reinforcement also eliminated the risk of sudden brittle fracture in the plywood due to the stress concentration caused by the inhomogeneous material properties.

Both an increase in the bending strength and a decrease in deflection were observed for the CFRP-reinforced samples. For the unreinforced samples, the maximum breaking load of 455 N was noted, while for the CFRP-reinforced samples, the maximum breaking load of 1050 N was noted. The modulus of elasticity (MOE) determined using the three-point bending method was 2200(±115) N/mm^2^ for the CFRP/plywood sample and MOE = 415(±35) N/mm^2^ for unreinforced plywood. The modulus of rupture, MOR, was 91(±12) N/mm^2^ for the CFRP/plywood samples and 32(±8) N/mm^2^ for the unreinforced plywood samples. The results are presented in Figure 5. As can be seen, for the CFRP-reinforced plywood sample, the value of the modulus of rupture was three times higher and the modulus of elasticity was more than five times higher than those for a plywood sample.

### 3.4. Deformation of Samples

**Figure 7:**
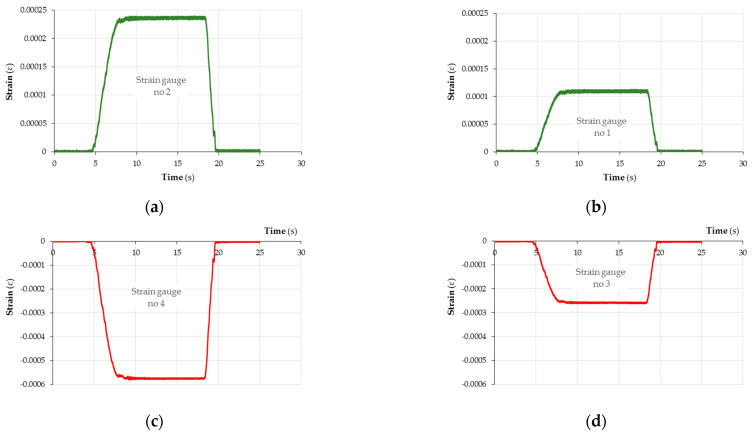
Deformation, ε, of CFRP-reinforced plywood sample measured using strain gauges (**a**) 2, (**b**) 1, (**c**) 4 and (**d**) 3.

**Figure 8:**
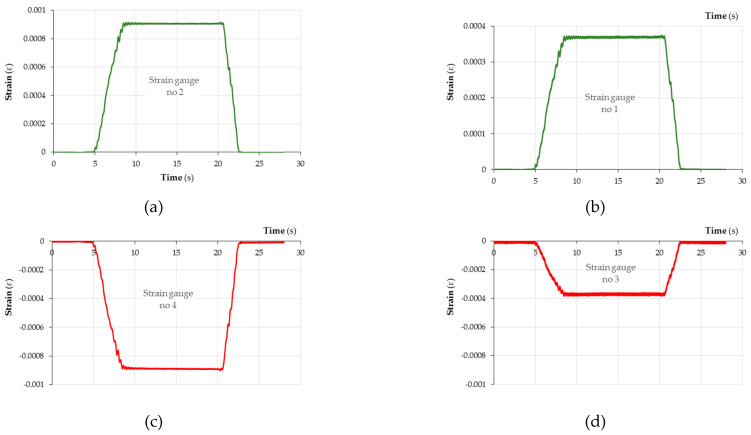
Deformation, ε, of unreinforced plywood sample measured using strain gauges (**a**) 2, (**b**) 1, (**c**) 4 and (**d**) 3.

Both plywood and CFRP panels are anisotropic materials, i.e., their mechanical properties vary in different spatial directions. The number of different directions of investigation is usually limited by the assumption of there being three mutually perpendicular planes. Strength tests for CFRP and plywood panels are rather common. However, there are few studies focusing on the behaviour of hybrid composites fabricated from a combination of CFRP and wood-based panels. Deformation measurements of solid wood and other materials are conventionally performed using strain gauges. Figure 7 and Figure 8 show the values of deformation, ε, of the CFRP-reinforced plywood sample and unreinforced sample. The location where the measurement of deformations on the sample surface was performed is shown in Figure A7. Based on the measurements of the sample deformations, ε, performed using the tensiometric method and based on the determined modulus of elasticity (MOE) (Figure 6) in the three-point bending test, the values of stresses occurring on the sample surface were determined. These stresses are of compressive and tensile characters.

**Figure 9:**
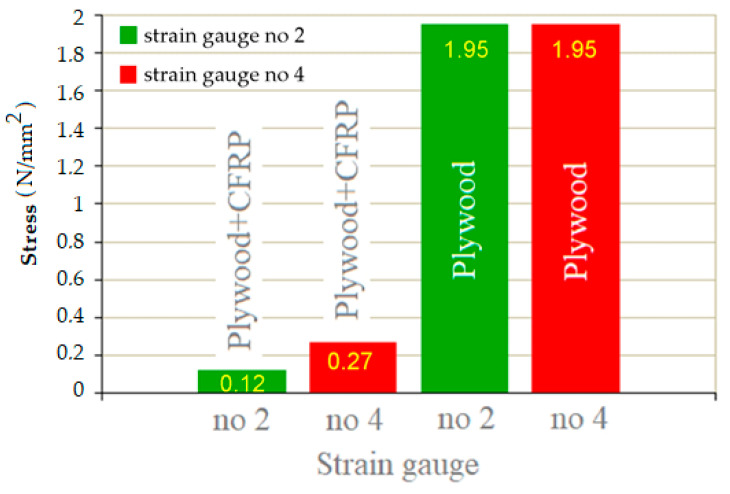
The values of tensile and compressive stresses obtained during measurements.

Figure 9 shows the stress values on the sample surfaces. The values of tensile (green bars) and compressive (red bars) stresses were obtained indirectly from measurements taken using strain gauges nos. 2 and 4 (Figure A7c). The stress values were determined using the simplified Hooke’s law according to Equation (5). Tensile stresses are marked in green, and compressive stresses in red. The CFRP-based reinforcement on the tensile side of the samples resulted in a reduction in both compressive and tensile stresses compared to the unreinforced sample. The application of a CFRP panel strengthened the plywood layers that were subject to tension by taking over the tensile stresses by the CFRP panel. This also resulted in a complete lack of brittle cracks in the plywood layers subjected to tensile stress. In the compressed zone of the CFRP-reinforced plywood, the stress values were reduced as a result of the limitation of strains by the introduced CFRP panel in the tensile area of the sample.

**Figure 10:**
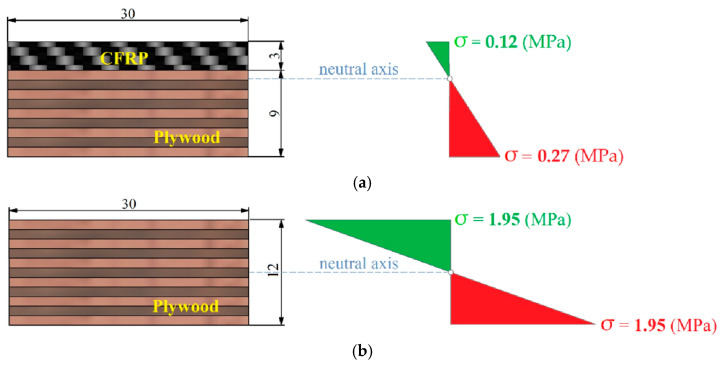
Comparison of stress distribution and neutral axis position: (**a**) CFRP-reinforced plywood panel; (**b**) plywood.

As can be seen in Figure 9 and Figure 10, in the case of using a CFRP-reinforced plywood panel, the value of tensile stresses was sixteen times lower (from 1.95 N/mm^2^ to 0.12 N/mm^2^), and the value of compressive stresses was more than seven times lower (from 1.95 N/mm^2^ to 0.27 N/mm^2^) than in the case of the unreinforced plywood sample. Moreover, the neutral axis of bending shifted towards the CFRP panel as a result of the hybrid structure of the analysed sample. The situation was completely different when analysing the bending of plywood samples. Similar stress values are noted on the sample’s surface regardless of the type of stress (tensile or compressive). For both types of stress, similar values were noted in the conducted tests. Due to the symmetry in the stress values, the position of the neutral axis passes through the geometric centre of the sample cross section.

The CFRP panel, despite having a smaller thickness, shows higher stiffness and strength compared to plywood. Through the adhesive joint, a high-strength CFRP panel relieves plywood, and consequently, the value of bending stresses is reduced. At the same time, the CFRP panel, being the outer layer, experiences lower stresses compared to unreinforced plywood.

### 3.5. System Dynamic Characteristics

**Figure A14:**
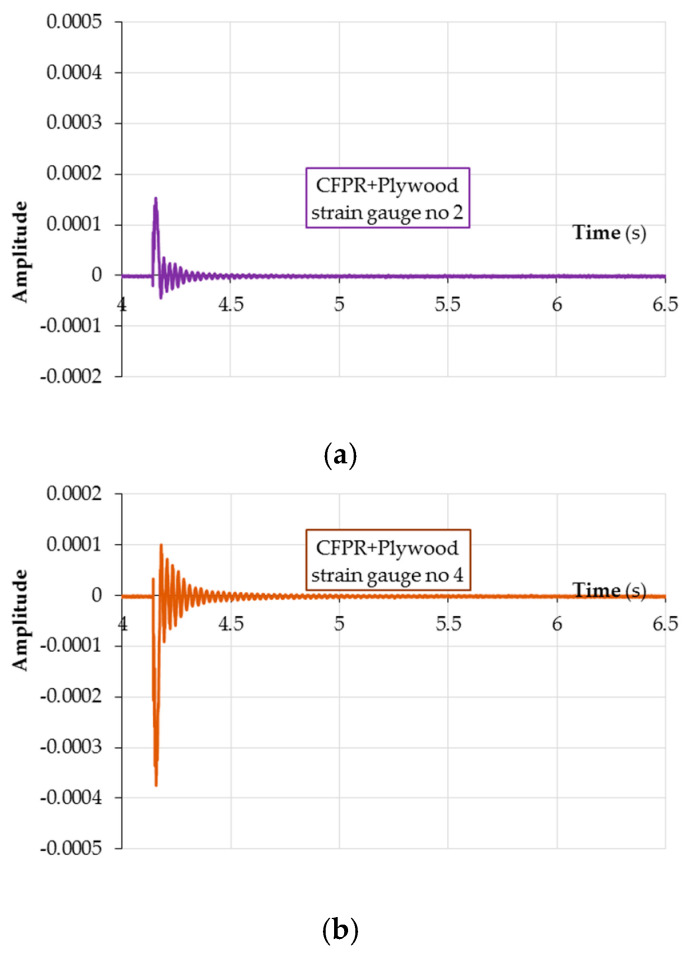
Time histories of strain amplitude of CFRP-reinforced plywood measured with (**a**) strain gauge no. 2 and (**b**) strain gauge no. 4.

**Figure A15:**
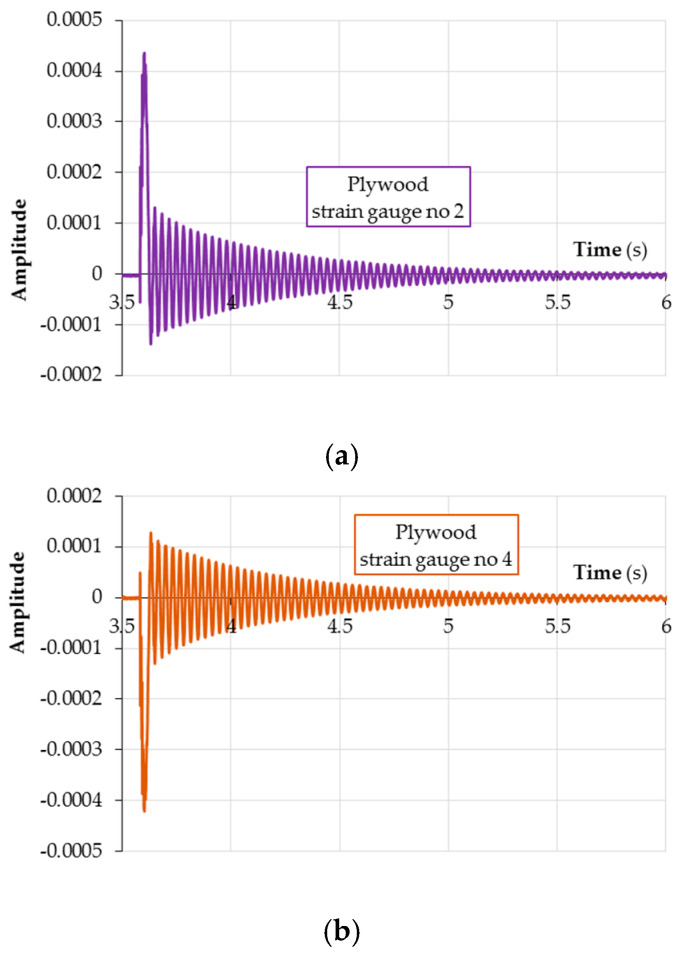
Time histories of strain amplitude of plywood measured with (**a**) strain gauge no. 2 and (**b**) strain gauge no. 4.

Figure A14 and Figure A15 show an example of the time histories of the amplitude of deflection from the equilibrium state of the tested materials. The strengthening of plywood with a CFRP panel significantly reduced the vibration amplitude (Figure A14) in relation to plywood (Figure A15). At the same time, there was a noticeably shorter vibration damping time (Figure A14).

**Figure A16:**
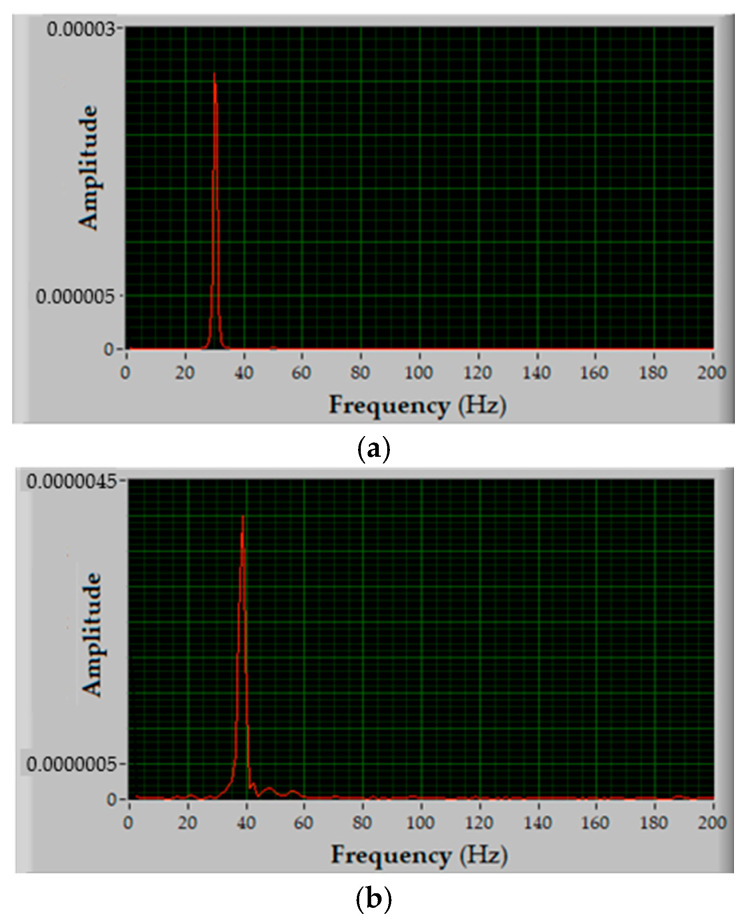
Natural frequency of cantilever beam: (**a**) plywood; (**b**) CFRP-reinforced plywood.

Based on the signal of the exciting force and the response of the system in the form of acceleration, a modal analysis was performed, which allowed for determining the natural frequency of the sample vibrations. The beginning of a single strike with a modal hammer was determined. It is recognised on the force signal as exceeding the threshold, which is five times the maximum value of the first 100 signal samples. Then, 750 samples are extracted [59]. For the applied sampling frequency of 50 kHz, the single hit window is 15 ms. Figure A15 shows sample spectra extracted from the entire signal using the above criteria. It was found that the frequency of natural vibrations of the CFRP-reinforced plywood samples increased by about 33% (from 30 Hz to 40 Hz) compared to the unreinforced plywood (Figure A16). Studies of the frequency of natural vibrations can help avoid resonance and design vibration isolation systems. It should be noted that the natural vibration frequencies and their corresponding vibration forms (modes) depend on the geometry of the component, material properties and mounting conditions. The difference in the natural frequency values between plywood and the CFRP-reinforced plywood samples results primarily from the combination of two materials with different density values. The higher density of the CFRP panel translates into an increase in the natural vibration values. The volume ratio in the hybrid composite panel, that is, one-quarter of the CFRP (3 mm) and three-quarters of plywood (9 mm), resulted in a 33% increase in the natural vibration values. The information provided in this article that the natural vibration frequency of CFRP-reinforced plywood increased by 33% is of comparative character (for a given case) in relation to plywood. This value cannot be applied to every developed geometry built using CFRP-reinforced plywood samples. This only allows us to state that the use of hybrid CFRP/plywood panels causes a larger change in the value of the natural vibration frequency than the value of the frequency of the force needed to excite the phenomenon of resonance changes.

When we know the value of the first natural frequency of the sample (ω_1_) and the geometry of the sample, we can determine the modulus of elasticity (MOE) from Equation (33). The modulus of elasticity determined using the dynamic method for CFRP-reinforced plywood samples was MOE = 2348 N/mm^2^. On the other hand, the value of the modulus of elasticity for plywood samples was recorded as MOE = 401 N/mm^2^. In the tests conducted to determine the modulus of elasticity, no statistically significant differences were observed in terms of the assessment method. In the tests conducted to determine the modulus of elasticity in the three-point bending test, the result was MOE = 2200 N/mm^2^ for the CFRP-reinforced plywood and MOE = 2200 N/mm^2^ for the plywood samples.

## 4. Conclusions

This paper presents the results of research conducted to determine selected strength parameters of plywood samples externally reinforced with a CFRP panel based on a three-point bending test, static tensile test and dynamic analysis. The influence of the CFRP panel’s application on the change in the natural vibration value of the obtained CFRP-reinforced samples in comparison with plywood was also assessed. The research results were compared with the theoretical analysis. Based on the results obtained, the following can be stated:

(1) All samples tested in the three-point bending process failed in the areas furthest from the neutral axis. In the case of unreinforced samples, the failure was abrupt and manifested as a sudden crack. In the case of CFRP-reinforced plywood samples, the failure process was preceded by a relatively slow propagation of cracks.
(2) During the bending tests, no failure of the adhesive bond between the CFRP panel and plywood was observed.
(3) Both an increase in the bending strength and a decrease in deflection were observed in the case of CFRP-reinforced plywood samples.
(4) For the CFRP-reinforced plywood, where the sample was subjected to tension stress, the value of tensile stress decreased by sixteen-fold (from 1.95 N/mm^2^ to 0.12 N/mm^2^), and the value of compressive stress decreased by more than seven-fold (from 1.95 N/mm^2^ to 0.27 N/mm^2^) compared to the unreinforced plywood sample.
(5) Based on the stress occurring at the tensile and compressive sides of the CFRP-reinforced plywood sample’s surface during a cantilever bending test, it was found that the value of the modulus of rupture (MOR) decreased by three-fold and the value of the modulus of elasticity (MOE) decreased by more than five-fold compared to the unreinforced plywood sample.
(6) A dynamic analysis allowed us to determine that the frequency of natural vibrations of the CFRP-reinforced plywood sample increased by about 33% (from 30 Hz to 40 Hz) compared to the unreinforced plywood sample.

In the tests conducted to determine the modulus of elasticity (MOE) of the two types of samples tested, no statistically significant differences were observed in terms of the method of their determination.

## Figures and Tables

**Table 1:** Selected mechanical and physical properties of 12 mm thick plywood panel and CFRP panel (values in parentheses are standard deviations).

Material	Density (g/cm^3^)	Modulus of Elasticity (N/mm^2^)	Bending Strength (N/mm^2^)	Ultimate Tensile Strength (N/mm^2^)
Plywood	0.652 (0.1)	5560 (261)	2200 (189)	36 (5)
CFRP	1.55 (0.3)	64,530 (676)	82,000 (645)	752 (25)

**Table 2:** Selected mechanical properties of ACRA LOCK SA 10-15 BLK methacrylic adhesive.

Ultimate Tensile Strength (N/mm^2^)	Hardness (Shore)	Apparent Shear Strength (N/mm^2^)
21–24	70 D	17–21

## Data Availability

The original contributions presented in this study are included in the article. Further inquiries can be directed to the corresponding author.
